# Key role in ecosystem functioning of scavengers reliant on a single common species

**DOI:** 10.1038/srep29641

**Published:** 2016-07-12

**Authors:** Richard Inger, Esra Per, Daniel T.C. Cox, Kevin J. Gaston

**Affiliations:** 1 Environment and Sustainability Institute, University of Exeter, Penryn, U.K.; 2 Faculty of Science, Biology Department, Gazi University, Teknikokullar, Ankara, Turkey

## Abstract

The importance of species richness in maintaining ecosystem function in the field remains unclear. Recent studies however have suggested that in some systems functionality is maintained by a few abundant species. Here we determine this relationship by quantifying the species responsible for a key ecosystem role, carcass removal by scavengers. We find that, unlike those within largely unaltered environments, the scavenger community within our highly altered system is dominated by a single species, the Carrion crow, despite the presence of a number of other scavenging species. Furthermore, we find no relationship between abundance of crows and carcass removal. However, the overall activity of crows predicts carcass biomass removal rate in an asymptotic manner, suggesting that a relatively low level of abundance and scavenging activity is required to maintain this component of ecosystem function.

The relationship between biodiversity and ecosystem function has been the subject of decades of intensive experimental research, and lies at the heart of key concepts in ecology and conservation biology. Experimental results overwhelmingly suggest that reduction in species richness leads to loss of ecosystem function^1^. These experiments tend however to be highly simplified with very small plot sizes and controlled abundances for each species. Hence how these findings reflect the relationship in natural ecosystems remains largely untested^2^. Whilst there is some emerging support for the importance of species richness in delivering real world ecosystem function^3^,^4^ it has been believed for some time that the most common species are likely to be disproportionately responsible^5^. Recent evidence supports this view. For example, crop pollination can be dominated by the activity of a few abundant species of bees^6^. Similarly, pest control via avian predation of arthropods in agroforestry plantations is mostly due to the activity of a single species of insectivorous bird, with species richness having no effect^7^.

Here we examine the relative importance of individual species in delivering a key component of ecosystem functioning, carcass removal by scavengers. Scavenging results in more energy being held in higher trophic levels and promotes the linkage of different (detrital & heterotrophic) food webs, which are key for ecosystem structure, function and stability^8^,^9^. Scavenging is more phylogenetically and geographically widespread than previously thought, with many predators, including taxa not previously thought to do so, actually being facultative scavengers^10^. Mobile scavengers redistribute energy and nutrients within ecosystems and across ecosystem boundaries^11^,^12^. A rapidly growing body of research has demonstrated that scavenging also provides an important ecosystem service role, the removal of carcasses from the environment, and the associated hygiene benefits^13^,^14^,^15^,^16^.

In this study we identify the species richness and proportional carcass removal activities of a scavenger community using camera traps on experimentally deployed and standardised carcasses.

## Results

**Figure 1:**
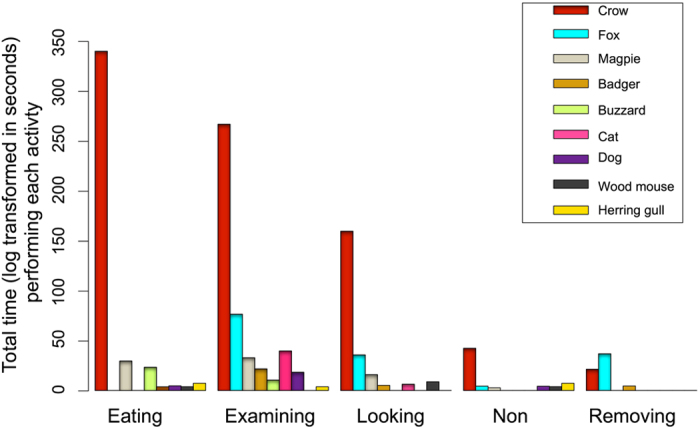
Total time spent in different activities by different scavenger species.

A total of 17 vertebrate species were recorded by camera traps at 63 (90%) of 70 deployed experimental rat carcasses. The total activity time was 113 hours, of which 94% was by Carrion crows (*Corvus corone*; Fig. 1). When only time spent eating the carrion was considered, 98% of the activity was by crows. Scavenger activity (eating or removing the carcass) was recorded at 49 carcasses (70%) by nine species. Vertebrate activity was recorded at 11 of our 12 study sites and scavenger activity was recorded at all 11 active sites. Seven species (Carrion crow, Common buzzard *Buteo buteo*, Domestic cat *Felis catus*, Domestic dog *Canis familiaris*, European magpie *Pica pica*, Herring Gull *Larus argentatus* & Wood mouse *Apodemus sylvaticus*) were recorded eating the carcass. Two species only ever removed the carcass (European badger *Meles meles*, Red fox V*ulpes vulpes*). One whole carcass was removed by a badger, and the remains of 9 carcasses were removed at night by foxes after originally being scavenged by crows. Crows were recorded both feeding on, and removing the remnants of a further five carcasses. Changes in carcass biomass differed considerably and significantly (R^2^_GLMM(m)_ = 0.40, R^2^_GLMM(c)_ = 0.50, F_[1,98]_ = 99.27, p < 0.001) between experimental (scavengers having full access) (mean loss = 175.70 g, SD = 119.40) and encaged (which scavengers could not access) control groups (mean loss = 16.34 g, SD = 38.02). Apparent species richness was also significant (F_[1,107]_ = 7.659, p = 0.007), with a general increase in biomass removed with increasing species richness, although the effect size in terms of the parameter estimate was considerably smaller than that of the experimental treatments (treatment β = 38.47 (SE = 4.48); apparent species richness β = 4.46 (SE = 1.61)).

The GLM to explore variation in activity time by species explained 70% of the variation (R^2^_GLMM(m)_ = 0.687, R^2^_GLMM(c)_ = 0.706), with almost all of this due to the fixed factors, indicating that there were negligible differences between the different experimental replicates or study sites as these were random factors in the analysis. We found significant differences in the time different species were observed around the carcass (F_[8,194]_ = 6.401, p < 0.001), and a significant interaction between species and behaviour (F_[17,192]_ = 3.186, p < 0.001), suggesting that different species were favouring different behaviours. This is overwhelmingly due to the much higher activity of crows, and that crows spent a greater proportion of their time feeding on the carcass (Fig. 1). Total time the carcass was deployed was not significant.

**Figure 2:**
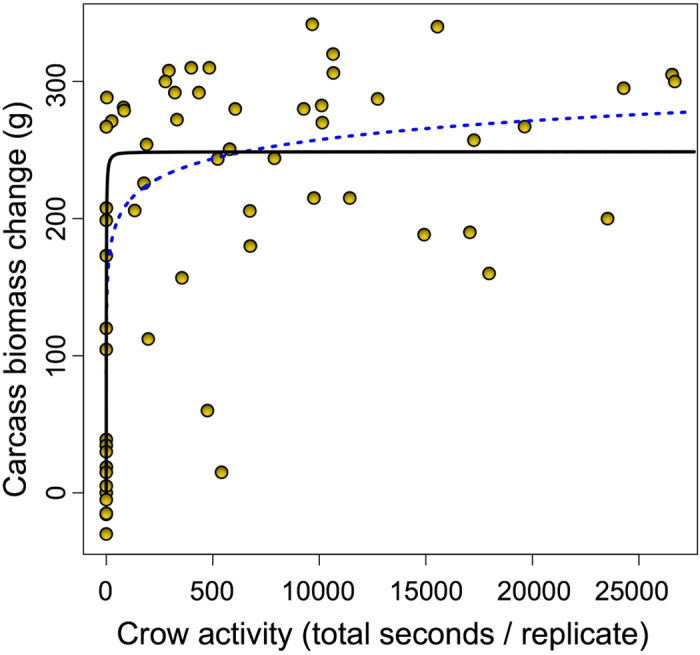
Relationship between crow activity and change in carcass biomass (Zero = no change in the carcass biomass during the course of the experiment). Black solid line is the fit from the Michaelis-Menten asymptotic model (RMSE = 74.4), blue dotted line is the fit from the exponential model (RMSE = 80.0, R^2^ = 0.49).

There was no significant relationship between abundance of crows and carcass biomass loss. There was however a positive relationship between crow activity around the carcasses and carcass biomass loss. All of the models were significant (p < 0.001) although the asymptotic model had the lowest RMSE (asymptotic = 74.4, exponential = 80.0, GAM = 82.1, polynomial = 95.5, linear = 101.1, Fig. 2). Currently it is not possible to calculate reliable coefficients of variation (R^2^) for non-linear models^17^ but the best fitting model for which value could be calculated, the exponential model, had a R^2^ of 0.49.

## Discussion

Despite a relatively rich scavenger community, and a positive relationship between carcass removal and species richness, overall carcass removal was dominated by a single species, the Carrion crow. Our results support recent findings suggesting that biodiversity, in terms of species richness, may not be necessary to maintain certain components of ecosystem functioning and the subsequent ecosystem services they provide. In this study we have quantified the first step in this process, the consumption of carcasses by scavengers. Clearly this can be considered an ecosystem service as removal of carcasses from the environment is beneficial to humans. What remains unclear is how this component of ecosystem functioning impacts other ecosystem processes and ultimately overall ecosystem function.

Nine species were identified as active scavengers, which is within the range (6–29 species, mean = 14) found in a wide variety of largely undisturbed habitats^10^. That the number of scavengers is lower than the global average is unsurprising given the highly modified, agricultural nature of our study system.

In natural habitats the scavenger community is generally highly structured and carrion resources are distributed fairly evenly throughout the community^10^,^18^,^19^. In more altered, large scale agricultural habitats, the competitive balance for carrion favours fewer, more generalist consumers^18^. Our study system consisting of small scale agriculture, sparse woodland fragments and interspersed rural human habitation will be even more fragmented, which may act to suppress the activity of some scavengers. This may help the highly mobile species, particularly those able to transmit information on carcass location socially, such as corvids^20^, to monopolise carcass resources. Hence habitat fragmentation may be a driving force behind the dominance of crows as scavengers. Indeed, this represents a clear opportunity for future research.

Of course species richness is only one aspect of biodiversity, of equal importance is the abundance of individuals within a population, and the levels of activities which contribute to ecosystem functioning^21^. Whilst the relationship between species richness and ecosystem function has received considerable attention, the role of abundance and the shape of the abundance versus functional delivery relationship remains largely unknown. Here we find no relationship between the abundance of crows and rates of carcass removal. However, carcass removal was directly and asymptotically related to the activity of crows, with a very sharp rise in rates of removal for a small increase in activity, suggesting that relatively low levels of activity are needed to maintain the ecosystem function, with little or no gain made after the asymptotic level of activity. Mechanistically this is likely due to competition over a relatively small carcass, with only a limited number of crows able to feed at any one time. Of course the shape of this relationship will likely be influenced by the density and size spectra of the carcass resource, both of which have rarely been quantified. Whilst our results demonstrate that abundance is not related to carcass removal rates in our system, we suspect that abundance will be important at a wider landscape level. For scavengers to be effective in their removal they must be able to search vast areas for carcasses (as is the case with vultures), or be abundant and widespread enough to locate carcasses.

Although we attempted to ensure our study replicated natural scavenging behaviour there are a number of limitation in the experimental design that could be addressed in the future. First, we only used a single carcass species and size, whereas different scavenger species may have preferences for different kinds of carcasses. Second. we only placed the carcasses in the field around midday, which may potentially be advantageous for diurnal scavengers.

Our findings build on recent work demonstrating the functional importance of the most abundant species in maintaining ecosystem function^5^,^6^,^7^,^18^. This is not to say that species richness is not important, particularly at wider spatial scales encompassing different environments. In this particular study system crows are clearly the primary scavengers, although this may not be the case in other habitats. Greater species richness should also provide resilience within ecosystems to habitat alteration^22^,^23^ or to the decline or loss of abundant species. Being abundant does not necessarily protect species from decline. For example, across Europe the most common bird species seem recently to have undergone the most rapid declines^24^, and historically there have been numerous cases of once common species being driven to extinction^5^. Whist the main ecosystem function provider in our study, the Carrion crow, is not in decline^24^, it is one of the most disliked bird species in the UK^25^ and can be legally killed as a pest species.

## Methods

We established 12 study sites within rural and agricultural environments within a 10 km radius of Falmouth, Cornwall, UK, between May and September 2015. Sites were generally well separated (>2 km), apart from 3 sites that were more closely clustered. Six replicate experiments were conducted at each site, apart from one site where, due to technical failures, only four replicates were possible (n = 70). Each replicate consisted of a commercially sourced rat carcass (250–300 g, Live Foods Direct, http://www.livefoodsdirect.co.uk), to be used as carrion, secured to a wooden board (45 cm × 30 cm) on the ground, and placed 2 m in front of a Reconyx HC 600 Hyperfire camera trap. Cameras were set to take 3.1 megapixel colour images by day and infrared monochrome images by night. Trigger speed was 0.2 seconds with a reset speed of approximately 0.5 sec, meaning the camera produced a ‘near video’ series of images of all animals attending the carcass. Rats were secured to the platform with two nails to ensure the carrion remained within the view of the camera trap in order to maximise the observations of scavenging behavior. At four of the replicates (n = 48) we also deployed control carcasses placed in wooden cages that allowed invertebrates but prevented vertebrates from gaining access. Carcasses were left in the field for 3–4 days (Mean = 3.37 days SD = 0.526) after which they were removed, weighed and data recovered. All carcasses were placed around midday.

### Photographic Analysis

Photos were examined sequentially for the presence of vertebrates. Each vertebrate recorded was assigned an activity relating to its interaction with the carcass: “None” - animal had no interaction with the carcass; “Looking” - animal looking in the general direction of the carcass; “Examining” - animal close to (approx. <2 rat lengths from) the carcass, looking/sniffing at the carcass; “Eating” - animal consuming the carcass; and “Removing” - animal removing the whole carcass from the experiment. Should the interaction change the new interaction was recorded as a new observation. Once the animal left the view of the camera we had no means of knowing if a subsequent visit by the same species was the same or a different individual, hence we have no information on the number of individuals of a particular species for any particular experiment. Time spent on each behaviour and the total time was calculated for each species and each replicate. Apparent species richness was also recorded as the number of different species captured by the camera trap for each replicate.

### Abundance and Activity of Birds

Preliminary surveys indicated that Carrion crows were responsible for a considerable amount of scavenging behaviour. Therefore, we produced estimates of crow activity and abundance to determine the shape of the relationship between abundance/activity and rates of biomass removal through scavenging. Total crow activity time (in seconds for all activities) per replicate was calculated from the photographic analysis. Crow abundance estimates for each site (birds/hectare) were calculated from point counts using a hierarchical distance sampling model^26^. Timed (10 min) counts were taken 4 times at each site within 10 meters of the experimental site, with bird number and their radial distance from the observer recorded into bands of 0–20, 20–40, 40–60, 60–100 and 100–200 m Abundance estimates were then calculated using the ‘unmarked’ package^27^ using the ‘distsamp’ function with a half normal detection function, with no covariates.

### Statistical Analysis

To identify differences in changes in carcass biomass between experimental and control carcasses we used a general linear mixed model (GLMM) with a Gaussian error structure, with rank transformed biomass change as the dependant variable, treatment (experimental carcass vs caged control carcass) as a fixed factor, days the carcass was deployed as a covariate, apparent species richness as a covariate, and site as a random factor. GLMMs were fitted using package ‘lme4’^28^. To examine differences in species and their behaviours around the carcass we used a GLMM with a Gaussian error structure with log transformed total activity (by species and replicate) as the dependant variable and behaviour, species, and the interaction between behaviour and species as fixed factors, site and replicate as random factors, and days deployed as a covariate. Residuals from models were checked to verify the error structure used. Variance explained was calculated using the methods of Nakagawa & Schielzeth^29^. We calculated R^2^_GLMM(m)_, the marginal R^2^ which describes the variance explained by the fixed factors, and R^2^_GLMM(c)_, the conditional R^2^ which is concerned with the variance explained by both the fixed and random factors. Degrees of freedom were estimated^30^, and p-values calculated for mixed effect models using the ‘lmerTest’ package^31^. To establish the relationship between crow activity/abundance and scavenging rates we fitted linear, polynomial, exponential, non-linear asymptotic and general additive models to the data. The best fitting model was judged to be that with the lowest root mean squared error (RMSE). All analysis was conducted in R (v3.0.2)^32^

## Additional Information

**How to cite this article**: Inger, R. *et al*. Key role in ecosystem functioning of scavengers reliant on a single common species. *Sci. Rep.* **6**, 29641; doi: 10.1038/srep29641 (2016).
